# Combinatorial engineering pinpoints shikimate pathway bottlenecks in para-aminobenzoic acid production in *Pseudomonas putida*

**DOI:** 10.1186/s13036-025-00553-5

**Published:** 2025-09-30

**Authors:** Marco A Campos-Magaña, Sara Moreno-Paz, Maria Martin-Pascual, Vitor AP Martins dos Santos, Luis Garcia-Morales, Maria Suarez-Diez

**Affiliations:** 1 Laboratory of Systems and Synthetic Biology, Wageningen University & Research, Wageningen, the Netherlands; 2 Laboratory of Bioprocess Engineering, Wageningen University & Research, Wageningen, the Netherlands; 3 LifeGlimmer GmbH, Berlin, Germany

## Abstract

Combinatorial expression libraries to optimize multigene pathways can improve product titers, but the large number of potential genetic variants makes exhaustive testing impractical. Statistical Design of Experiments (DoE) offers a powerful alternative to enable efficient exploration of gene expression landscapes with a limited number of measurements. Here, we applied this approach to modulate expression levels across all genes in the shikimate and para-aminobenzoic acid (pABA) biosynthesis pathways in *Pseudomonas putida*. From a theoretical library of 512 strain variants, we trained a regression model using a statistically structured sample comprising 2.7% of the total library, as defined by our DoE approach, and used the model to predict new genotypes with improved pABA titers. This strategy enabled us to achieve product titers ranging from 2 to 186.2 mg/L in the initial screen and subsequently guide a second round of strain engineering, culminating in a maximum titer of 232.1 mg/L. Our analysis indicated that *aroB*, encoding 3-dehydroquinate synthase, is a critical bottleneck in pABA biosynthesis. This study highlights the utility of combining DoE with linear regression modeling to systematically optimize complex metabolic pathways, paving the way for more efficient microbial production.

**Supplementary Information:**

The online version contains supplementary material available at 10.1186/s13036-025-00553-5.

## Introduction

Industrial biotechnology uses microbial cell factories as an alternative to the petroleum-based chemical industry, a sector with the third largest greenhouse gas emissions in industry [1]. Specifically, the shikimate pathway, responsible for the synthesis of aromatic amino acids, is considered a potential source of relevant compounds including polymer precursors (cis, cis-muconic acid), food additives (tryptophan), pharmaceuticals (salicylic acid), aromatic agents (vanillin) and biofuels (2-phenylethanol) currently produced via chemical conversion from petroleum-derived benzene, toluene, and xylene [2, 3]. This metabolic pathway has been exploited for the microbial production of numerous highly valuable compounds. For example, the ShikiFactory100 project focused on the engineering of microbial chassis to optimize the production of a number of shikimate pathway-derived compounds [4] including resveratrol [5] psilocybin [6] and *p*-coumaric acid [7] among others, underscoring its significant potential. They also described for the first time the microbial production of chavicol and eugenol from phenylacrylic acid substrates [8] and the production rosmarinic acid production from glucose in mineral media [9].

**Fig. 1:**
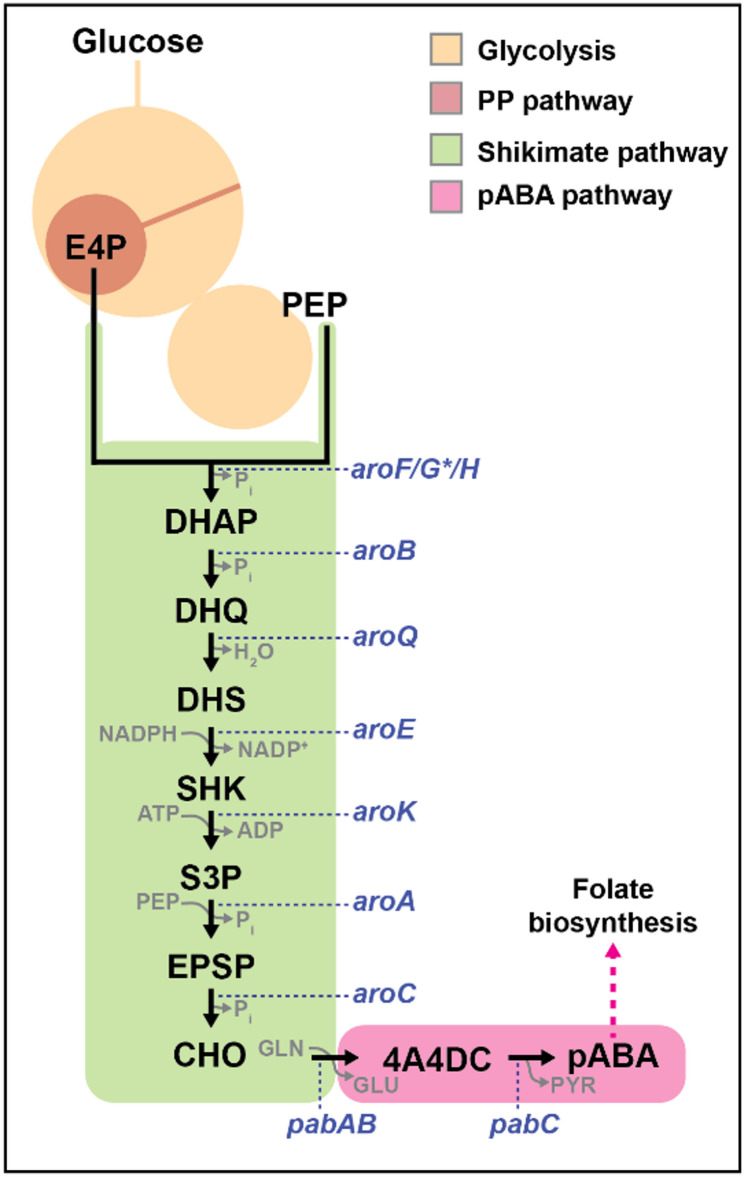
Biosynthetic pathway of pABA from glucose. Simplified map of the metabolic pathway for the production of pABA, which is derived from metabolites produced via glycolysis, pentose phosphate pathway, and the shikimate pathway. PP pathway: Pentose Phosphate pathway; E4P, erythrose 4-phosphate; PEP, phosphoenolpyruvate; P_i_, phosphate; DAHP, 3-deoxy-D-arabino-heptulosonate 7-phosphate; DHQ, 3-dehydroquinate; DHS, 3-dehydroshikimate; SHK, shikimate; S3P, shikimate 3-phosphate; EPSP, 5-enoylpyruvoyl-shikimate 3-phophate; CHO, chorismate; 4A4DC, 4-amino-4-deoxychorismate; pABA, *p*-aminobenzoic acid. *AroF/G/H*, 3-deoxyarabinoheptulosonate synthase; *AroB*, 3-dehydroquinate synthase; *AroQ*, 3-dehydroquinate dehydratase; *AroE*, quinate/shikimate dehydrogenase; *AroK*, shikimate kinase; *AroA*, 3-phosphoshikimate 1carboxylvinyl transferase; *AroC*, chorismate synthase; *PabAB*, aminobenzoate synthase; *pabC*, 4amino-4-deoxychorismate lyase. Enzymes subject to feed-back regulation are marked with *^3,10^

The shikimate pathway starts with the condensation of erythrose 4-phosphate and phosphoenolpyruvate to form 3-deoxy-D-arabino-heptulosonate 7-phosphate, which, through a series of six reactions, is converted into chorismate (Fig. 1). Chorismate can then be converted into the three aromatic amino acids, phenylalanine, tyrosine, and tryptophan, as well as serving as a precursor for other aromatic compounds [10]. However, obtaining high titers of aromatic compounds is challenged by the supply of precursors, the presence of regulatory systems, and cytotoxicity [3]. The tight regulation of the shikimate pathway results in low metabolic fluxes that hinder product formation. Although a common approach to increase the carbon flux towards production is the over-expression of shikimate pathway genes, there is no agreement on which genes to over-express for each specific product or microbial host. For instance, production of the shikimate-derived product protocatechuic acid in *Pseudomonas putida* benefited from the over-expression of 3-dehydroquinate dehydratase (*aroQ*) which converts 3-dehydroquinate to 3-dehydroshikimate [11]. In *Escherichia coli* the over-expression of 3-phosphoshikimate-1-carboxylvinyl transferase (*aroA*), which converts shikimate 3-phophate to 5-enoylpyruvoyl-shikimate 3-phophate, has shown beneficial effects for the production of coumarins, and salvianic acid, while over-expression of 3-dehydroquinate synthase (*aroB*) that converts 3-deoxy-D-arabino-heptulosonate 7-phosphate to 3-dehydroquinate, shikimate dehydrogenase (*aroE*) that converts 3-dehydroshikimate to shikimate, and shikimate kinase (*aroK*) that converts shikimate to shikimate 3-phosphate were specific for the production of violacein, salvianic acid, and tyrosine, respectively [10, 12]. Alternatively, over-expression of *aroA* and *aroD*, homologs of 3-deoxyarabinoheptulosonate synthase (*aroG*) that converts erythrose 4-phosphate (E4P) and phosphoenolpyruvate (PEP) to 3-deoxy-D-arabino-heptulosonate 7-phosphate and *aroE*, led to an enhanced production of shikimate in *Bacillus subtilis* [13].

Here, we use statistical design of experiments (DoE) to explore the effect of the expression of shikimate pathway genes on product formation while minimizing the number of strains to construct and test. As opposed to one-factor-at-a-time experimentation, which would require testing the effect of individual gene over-expressions, DoE uses orthogonal designs that allow the identification of the effect of individual genes when they are over-expressed in combinations. In this way, interactions among genes, such as synergistic effects of simultaneous over-expressions can be detected [14–18]. The genes to over-express in each strain are selected to prevent confounding of individual gene effects. Multiple DoE designs are available [14, 18]. Among the different DoE designs, we used Plackett Burman [19] design to explore the impact of the modulation of levels of over-expression of all genes in the shikimate pathway on product accumulation. This design is based on orthogonal matrices for the efficient screening of factors. This orthogonality enables to estimate each of the main effects of the factors independently of the others [17, 18, 20]. However, the ability of a two-state Plackett Burman design to reveal specific synergistic effects is minimal. Once the strains are constructed, their production data is used to train a linear model in which each factor (i.e. gene) is associated to a model coefficient that determines the effect of the gene on production. Finally, an analysis of variance (ANOVA) is employed to identify genes with a significant positive or negative effect on product titer [14, 18]. It should be noted that we have used a variant of the Placket Burman method described in Lawson [14] and implemented in Grömping [21]. Although the origin of this design is Hadamard [22] we will keep using the Plackett Burman denomination as that has become widely used in the field. We used production of *p*-aminobenzoic acid (pABA) in *P. putida* as a case study for the application of DoE to identify genes in the shikimate pathway limiting production. Improving the efficiency of the shikimate pathway in *P. putida* is important due to its central role in the biosynthesis of aromatic compounds. *P. putida* has been postulated as a microbial chassis with inherent stress-resistance capabilities and high-levels of NADPH, a cofactor essential for the biosynthesis of shikimate pathway- derived compounds [2, 23–25]. In bacteria, pABA is synthesized from chorismate by the action of three enzymes: PabA, PabB, and PabC (Fig. 1) [2]. pABA is a precursor for the formation of folate and it also serves as a precursor for the pharmaceutical industry and as a crosslinking agent for the synthesis of resins and dyes [26, 27].

The PB design was chosen for its efficiency in screening a large number of variables with a minimal number of experiments, which was essential given the combinatorial complexity of our system (9 genes × 2 expression levels = 512 possible combinations). Constructing and testing all combinations would have been impractical. This design allowed as to estimate the individual effect of genes on pABA production while assuming negligible interaction effects, which is appropriate for an initial screening phase. We reduced the experimental burden as this strategy initially required only 16 strains. Other design approaches are also available (resolution III or IV) but those would have resulted in a higher number of constructs which we deemed less efficient for the initial screening phase.

## Materials and methods

### Promoter and vector backbone selections

To define the high and low states for the genetic variables (promoter strength, RBS strength, and plasmid copy number), we selected genetic parts from a characterized library of synthetic promoters and ribosome binding sites that covers a 72-fold dynamic range of gene expression in *P. putida* using the mNeonGreen fluorescent reporter [28]. Specifically, for the high-state, we selected JE111111, the strongest characterized promoter in the library and for the low-state, we did not choose the weakest from the library, but selected JE151111, a promoter providing a moderate-level fluorescence that could be detected and still achieve a quantifiable difference compared with the high-level promoter. Similarly, for the ribosome binding site (RBS), we selected JER04, the strongest characterized RBS in the library, as the high-state. For the low-state condition, we selected JER10, a RBS that provides a moderate-level fluorescence but is not the weakest from the library. JER10 exhibits approximately 37-fold lower expression relative to JER04. Moreover, we selected origins of replication showing a quantifiable difference between the high- and low-state from a study developed in our group [29]. For the high-state, we selected pSEVA231 (origin of replication pBBR1 with copies = 30 ± 7), a medium-copy number plasmid characterized in *P. putida* using sfGFP reporter that showed a medium-level fluorescence, and for the low-state, we selected pSEVA621 (origin of replication RK2 with copies = 20 ± 10), a low-copy number plasmid characterized in *P. putida* using sfGFP reporter that showed the least sfGFP fluorescence [29, 30]. These selections were guided by the need to achieve a significant and quantifiable difference between the high and low states while aiming that expression remained within a biologically functional range for *P. putida*. Plasmids pSEVA231 and pSEVA621 conferring resistance to kanamycin and gentamycin, respectively, were used for strain construction.

### Strain construction

All primers used in this study are listed in Table S1. All plasmids, strains, and gene sequences used in the present study are listed in Table S2, S3, and S4, respectively. Plasmids were built from individual genetic parts, comprising vector backbone, synthetic promoter, and enzyme coding genes with ribosome binding sites (RBS) and terminator (Fig. 1). For low gene expression level, pSEVA621, JE151111, and JER10 were used as backbone, promoter, and RBS, respectively. For the high gene expression level, pSEVA231, JE111111, and JER04 were used as backbone, promoter, and RBS, respectively. All three para-aminobenzoic genes (the complete ORFs) were amplified from genomic DNA of *E. coli* K-12. Shikimate pathway genes including *aroK*, *aroE*, *aroB*, *aroQ*, *aroA*, and *aroC* were amplified from genomic DNA of *P. putida* KT2440. *aroG* was amplified from *E. coli* K-12 genomic DNA using primers that introduced a mutation to generate the aroG^D146N^ alelle. All DNA fragments were amplified using Q5^®^ Hot Start High-Fidelity DNA Polymerase (New England Biolabs). DNA fragments were purified with NucleoSpin™ gel and PCR clean-up kits (Macherey-Nagel, Germany). Synthetic promoters were ordered from IDT (Integrated DNA Technologies). DNA assembly of the different constructs was performed using Golden Gate or Gibson assembly. All plasmids were transformed by heat shock in chemically competent *E. coli* DH5*αλ* pir and selected on LB agar with corresponding antibiotics. Colonies were screened through colony PCR with Phire Hot Start II DNA Polymerase (Thermo Fisher Scientific) using the screening primers listed in the Table S3. Plasmids were verified using Sanger sequencing (Macrogen inc.) or whole plasmid sequencing (Plasmidsaurus inc.) and subsequently transformed into *P. putida* KT2440 via electroporation. For this, 100 ng of plasmid was electroporated into 100 µl cell suspension aliquots with a voltage of 2.5 kV, 25 µF capacitance, and 200 Ω resistance [31].

### Bacterial strains and growth conditions

*P. putida* KT2440 and *E. coli* cultures were incubated at 30 °C and 37 °C respectively. For cloning purposes, both strains were propagated in Lysogeny Broth (LB) medium. For the preparation of solid media, 1.5% (w/v) agar was added. Antibiotics to select colonies harboring plasmids were used at the following concentrations: kanamycin (Km) 50 µg/ml and gentamycin (Gm) 10 µg/ml. All growth experiments were performed using M9 minimal medium (per liter; 3.88 g K_2_HPO_4_, 1.63 g NaH_2_PO_4_, 2.0 g (NH_4_)_2_SO_4,_ 10 mg ethylenediaminetetraacetic acid (EDTA), 0.1 g MgCl_2_·6H_2_O, 2 mg ZnSO_4_·7H_2_O, 1 mg CaCl_2_·2H_2_O, 5 mg FeSO_4_·7H_2_O, 0.2 mg Na_2_MoO_4_·2H_2_O, 0.2 mg CuSO_4_·5H_2_O, 0.4 mg CoCl_2_·6H_2_O, 1 mg MnCl_2_·2H_2_O, pH 7.0). Strains were precultured overnight in 10 ml LB with corresponding antibiotics. pABA production experiments were performed with 12 ml of M9 minimal medium supplemented with 70 mM glucose and corresponding antibiotics. Cells were grown for 48 h at 30 °C and 250 rpm in 50 ml mini-bioreactor tubes (Corning) in an Innova 44 incubator (New Brunswick Scientific). At the end of the cultivation, OD_600nm_ measurements were performed and cultures were centrifuged at 4700 g for 10 min. A volume of 0.5 ml of supernatant was used for pABA quantification. Production of pABA was measured using three colonies as biological replicates to evaluate variations for each experiment.

### Analytical methods

Cell growth was determined by measuring the optical density at 600 nm (OD_600nm_) using an OD600 DiluPhotometer spectrophotometer (IMPLEN). pABA titer was determined using HPLC (Shimadzu) with a C18 column (4.6 mm × 250 mm) and a UV/vis detector set at 235 nm. The mobile phase consisted of Milli-Q water (A), 100 mM formic acid (B), and acetonitrile (C) with a flow rate of 0.75 ml/min at 30 °C. Chromatographic separation of analytes was achieved using the following gradient program: t = 0–5 min: the mobile phase composition was held isocratic at A-80%, B-10%, and C-10%; from t = 5 to 12.5 min, a linear gradient was applied from A-80%, B-10%, C-10% to A-0%, B-10%, C-90%; followed by an isocratic hold from t = 12.5 to 15 min at A-80%, B-10%, and C-10%.

### Experimental design and statistical analysis

R (version 4.3.3) was used for the generation of the design and data analysis. Plackett Burman (PB) designs were generated with the ‘pb’ function from the FrF2 R package [21]. Using the pb() with the argument nfactors = 9, returns the first 9 columns of a 16-run Hadamard matrix [22]. This matrix can accommodate up to 15 factors, and since we specified 9 factors, only 9 were returned [32] with no dummy variables included for the unassigned columns [33]. The complete 9-column design matrix is included in the supplementary materials. Experimental data was used to train a linear model by least square linear regression using the lm R function. The summary function was used to obtain the ANOVA table which provides the estimated coefficients and their associated *p*-values that were corrected to account for multiple testing using Bonferroni. The adjusted coefficient of determination (Adj R [2]) was used to assess the model fit to experimental data. Differences in pABA concentration among experiments during the second round of strain engineering were evaluated by two-tailed unpaired t-tests. To further evaluate the approach, we selected a design matrix with 3 dummy variables. We repeated the design with 16 runs and specified 12 factors. The first 9 correspond to the ones under study and the 3 new ones correspond to the dummy variables.

## Results

### Identification of factors affecting pABA production

The synthesis of pABA from erythrose 4-phosphate and phosphoenolpyruvate requires 10 genes involved in nine enzymatic reactions: seven reactions from the shikimate pathway and two committed reactions for pABA production (Fig. 1). Therefore, we selected all the genes required for the synthesis of pABA as candidates for over-expression to optimize production. We used *aroB*, *aroQ*, *aroE*, *aroK*, *aroA*, and *aroC* genes from *P. putida* and the feedback-resistant variant *aroG*^D146N^ ,*pabA*,* pabB*, and *pabC* from *E. coli* as targets for over-expression.

**Fig. 2:**
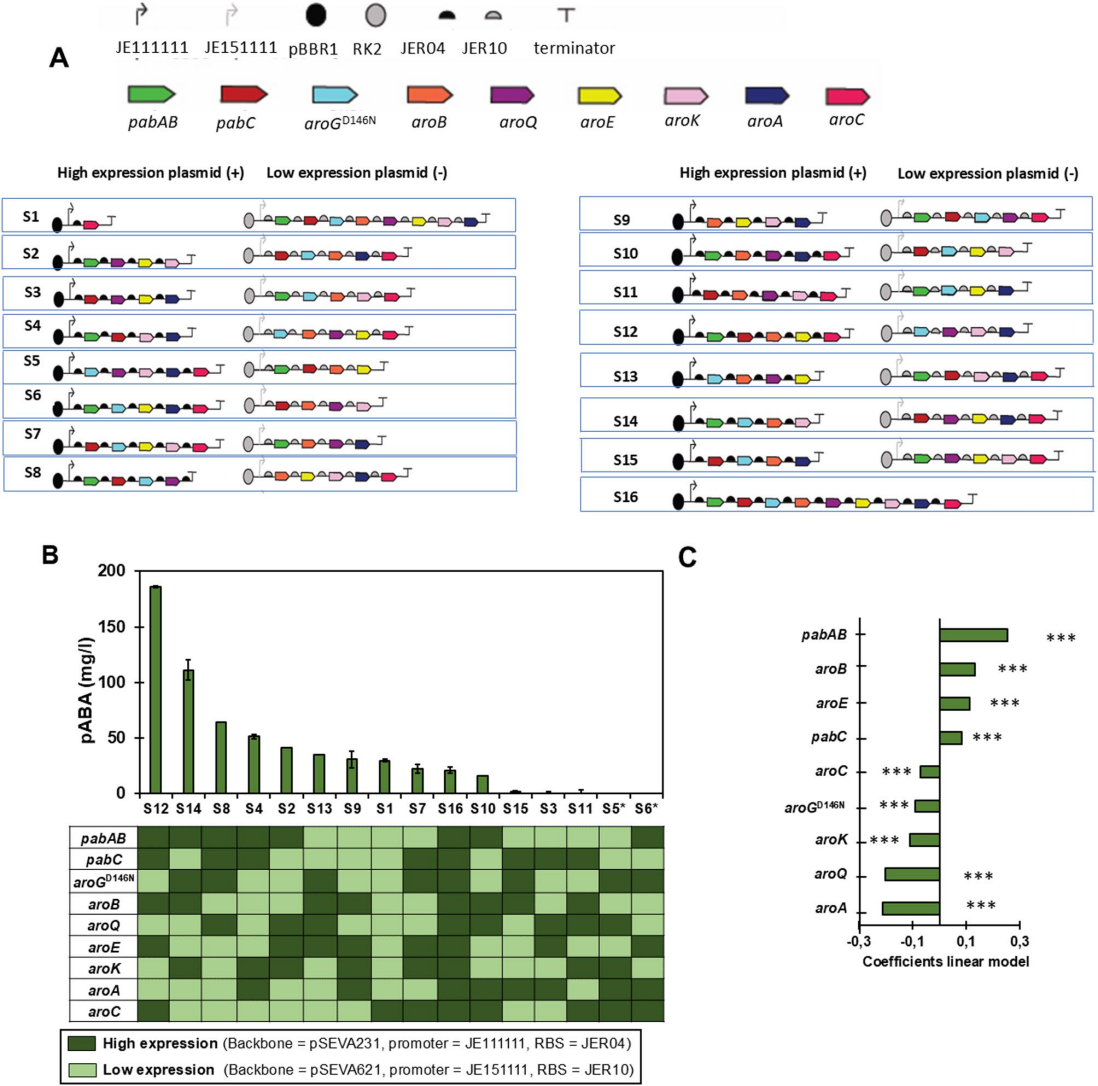
Identification of factors affecting pABA production. **(A)** Genetic constructs used for strains S1 to S16. Strains S1–S16 represent the 16 design points in the Plackett–Burman (PB) screening. Strains S1-S15 harbors a two-plasmid system (high-expression and low-expression plasmid). Strain 16 harbors a single-plasmid system (high-expression plasmid). Individual genetic parts used for high and low over-expression plasmids are listed at the top of the figure including a strong promoter (JE111111), weak promoter (JE151111), origin of replication (pBBR1), origin of replication (RK2), strong RBS (JER04), weak RBS (JER10), and terminator. Besides, genes used in the constructs are also listed at the top of the figure including *pabABC*, *aroG*^D146N^, *aroB*, *aroQ*, *aroE*, *aroK*, *aroA*, and *aroC*, Production is affected in each strain by a high over-expression plasmid (left) and low over-expression plasmid (right). **(B)** pABA production in strains S1 to S16 after 48 h culture in minimal media. Note that strains S5 and S6 could not be constructed and they are indicated with *. Values are means of the biological replicates, and the error bars indicate the standard deviations of all (*n* = 3) biological replicates. The heatmap represents the expression level for each gene (row) in each strain (column) based on whether it was placed on the high- or low-expression plasmid. Shades of green reflect the level of expression (light green for low, dark green for high), determined by the plasmid copy number, promoter, and RBS combination. **(C)** Regression coefficients of the linear model trained with data from B, adjusted R^2^ = 0.94. All the coefficients are significant according to ANOVA with *p*-values < 0.05 corrected using Bonferroni

We used a Plackett Burman design to explore the effect of gene over-expressions on pABA production. In this design combinations of genes are simultaneously over-expressed so the effect of each gene over-expression on pABA production can be determined [17, 20]. We considered each of the overexpression target genes as a single factor except for *pabA* and *pabB* which were considered together since the proteins coded by these two genes form a dimer complex [34] and therefore their unbalanced expression was expected to negatively impact pABA production [34]. Each of the factors was explored at two levels including moderate and high over-expression based on copy number plasmid, strength of the promoter, and ribosome binding site assigned to each gene. While studying the combinatorial effect of nine factors and two levels would require the construction of 512 strains (2^9^), the Plackett Burman design reduced the number of strains to build to 16 (2^4^), represented in Fig. 2A.

Fourteen of the sixteen strains required for the design were successfully constructed. Colonies with correct DNA assemblies for strains S5 and S6 were not found (Fig. 2A). PB designs are based on orthogonal matrices, which allow the estimation of main effects independently. The design we used was robust to missing data points because the effect of each gene was represented across multiple strain combinations. The absence of two strains, in this case, did compromise the balanced orthogonality of the design (Figure S7). However, the design still allows for estimation of main effects with minimal bias for the purpose of screening. This reinforces the value of DoE approaches such as PB, which can still yield actionable insights even when some designs are not experimentally feasible. The pABA production data measured with the available strains was sufficient to train a linear model (Adj R^2^ = 0.94) and obtain estimates of the coefficients for each of the tested factors (Fig. 2C). Across the different strains, pABA production exhibited a two-order-of-magnitude variation, ranging from 2.0 ± 3.4 mg/l to 186.2 ± 0.32 mg/l, demonstrating the effect of changing the expression levels of the selected genes on pABA production (Fig. 2B). The ANOVA on the linear model coefficients reported a significant effect of all the factors on pABA production with *p*-values < 0.05 corrected using Bonferroni (Fig. 2C) *p*-values from ANOVA are reported in Table 1. Estimated coefficients and their associated *p*-values were corrected to account for multiple testing using Bonferroni. The high over-expression of *pabAB* had the highest positive effect on pABA production, followed by *aroB*, and *aroE*. Therefore, a high expression of the *pabAB*, *aroB*, and *aroE* genes is essential to obtain high pABA titers. A weaker effect was observed for *pabC*. In addition, we identified genetic factors with negative regression coefficients, and therefore, a negative effect of high over-expression on pABA production. *AroA* and *aroQ* had the highest negative effect on pABA production. They were followed by *aroK*, *aroG*^D146N^, and *aroC* (Fig. 2B).

Notably, the expression strength levels of the genes in strain S12, the measured strain with the best titer, corresponded with the sign of all the estimated coefficients except for *aroC* (Fig. 2). The negative regression coefficients of the linear model suggest that overall high gene over-expression negatively affects pABA production as confirmed by strain S16.

We introduced 3 dummy variables into the regression model as internal negative controls and built again a linear regression model. As expected, the magnitude of the regression coefficients of the dummy variables were smaller than the effect of the genes, consistent with them being uninformative. Coefficients for two of the three dummy variables were flagged as statistically significant, indicating the coefficients are statistically distinguishable from zero in the model. These have values − 0.109 and 0.080 with standard errors 0.027 and 0.024 respectively, as can be seen in Table S2. These values serve as a reference to evaluate the magnitude of random variation within the current design. Coefficients obtained for the real factors have magnitudes in the 0.19 to 0.35 range and similar standard errors (see Table S2), which further highlights they are not the result of random noise.

We selected strains S1 and S16 to evaluate expression values using RT-qPCR and to consider the impact of operon length on the expression level of genes at the 5’ end since these two strains contain the largest operons from the design implemented in this study. Moreover, strain S1 contains all genes but *aroC* under a low expression-level and strain S16 contains all genes under a high expression-level and thereby, they can used to compare expression differences in all genes but *aroC* between strain S1 and S16. The results of this verification experiment indicated gene expression differences between strain S1 and S16 in agreement with the design specifications. In particular, strain S16 showed a higher expression than strain S1 for all the genes under a high-level of expression (Figure S6). Although the expression level of genes at the end of the operon was approximately 4-fold lower than that of the gene at the start of the operon, all genes at high-level state in strain S16 exhibited a higher expression than genes at low-level in the strain S1, suggesting the gene expression differences that were intended between a high-level from a low-level state were in fact achieved.

### Optimization of pABA production by expanding the design space

**Fig. 3:**
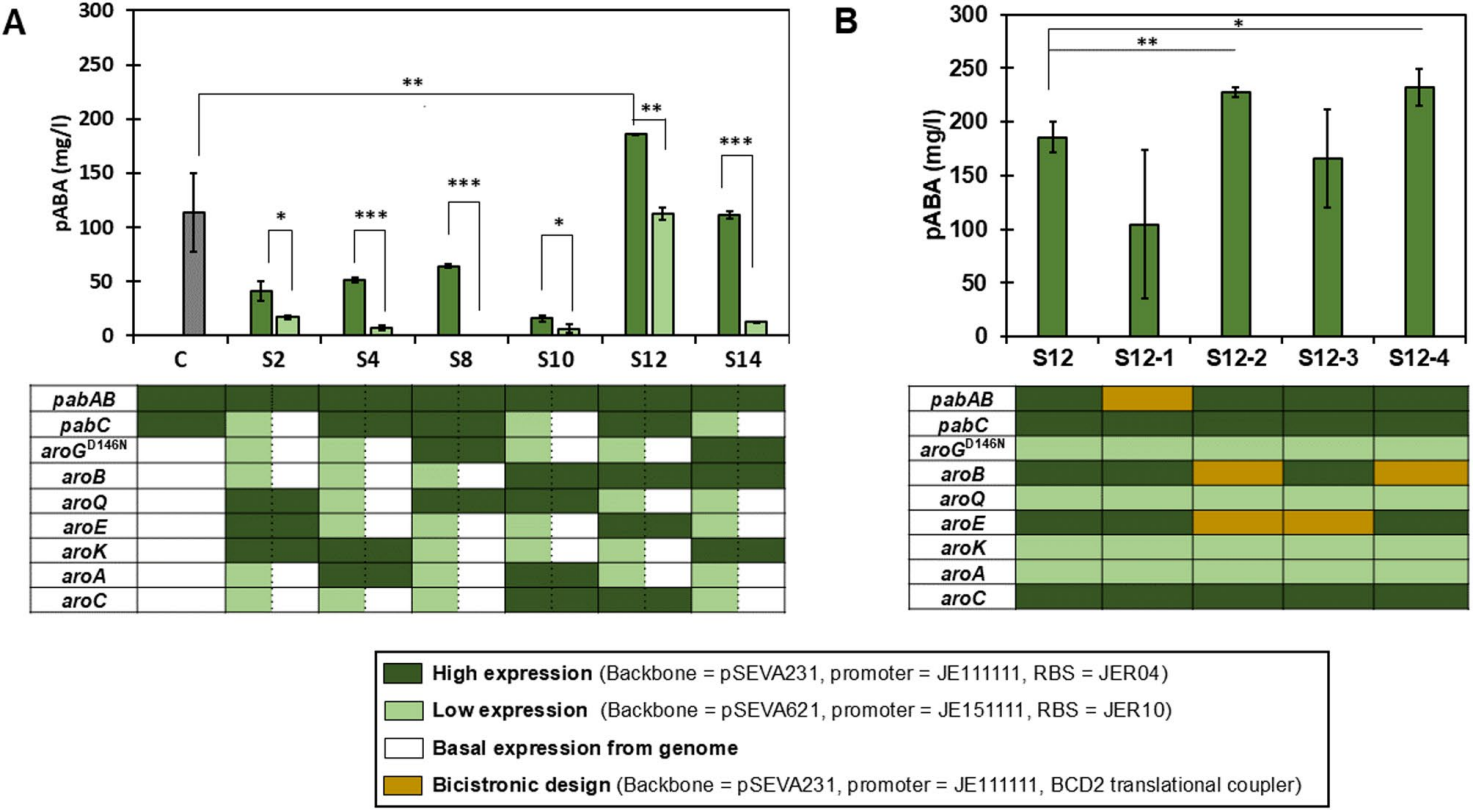
Expansion of the design space. **(A)** Effect of reducing gene over-expression to native expression levels on pABA production. Dark green bars represent strains S2, S4, S8, S10, S12, and S14 from Plackett Burman design (S1 to S16), while light green bars represent the strains S2, S4, S8, S10, S12, and S14 under the effect of reducing gene over-expression to basal/endogenous activity for the genes indicated in white cells. C denotes a control strain only over-expressing *pabABC.* The heatmap represents the expression level for each gene (row) in each strain (column) based on whether it was placed on the high-expression plasmid, low-expression plasmid or basal/endogenous activity. Shades of green reflect the level of expression (light green for low, dark green for high), determined by the plasmid copy number, promoter, and RBS combination. Shades in white represent basal/endogenous activity. **(B)** Effect of controlling expression levels using bicistronic designs on pABA production. Factors with BCD2 are indicated in yellow cells. The BCD2 translational coupler is inserted between the promoter and gene of interest. Values are means of the biological replicates, and the error bars indicate the standard deviations of all (*n* = 3) biological replicates. *P*-values were determined by two-tailed unpaired t-tests. *p < 0.05; **p < 0.01; ***p < 0.001

After identifying the effect of different degrees of over-expression of the tested genes, we intended to further improve pABA titers by testing a broader range of expression levels. First, the effect of native gene expression was compared to mild over-expression excluding high over-expression that resulted in low pABA titers. The mild over-expression level in the top producer strain S12, S14, S8, S4, and S2 (Fig. 3A) was used for this purpose. Additionally, we used bicistronic designs (BCD) as gene expression enhancers for the genes whose over-expression had a positive effect on pABA titers. A BCD enables to control protein translation by limiting interaction of mRNA secondary structures across 5’-unstranslated regions leading to a higher translational efficiency of the target gene [35–37]. In specific, we used the unit BCD2, a known translational coupler that has been described as being highly efficient in *E coli* and *P. putida* [36].

High over-expression of *pabAB* was identified as the factor with the highest positive influence on pABA production. Therefore, strains with these genes in the high-expression plasmid were used as background to study the effect of basal expression of the other genes. Additionally, a strain only over-expressing *pabABC* was used as control (C) to evaluate the impact of over-expressing shikimate pathway genes. In all cases, reducing gene expression from low over-expression to basal expression had a significant negative effect on pABA production (Fig. 3A; Table 1), indicating that, even if high over-expression is detrimental, mild over-expression is beneficial for production. The control strain also showed lower pABA titers compared to S12 showcasing that the over-expression of *pabABC* is not enough to obtain high pABA titers.

Bicistronic designs were used to study whether higher expression of *pabAB*, *aroB*, and *aroE*, the genes with the highest positive impact on pABA production, could further improve pABA titers. While the BCD was introduced in the genes with the highest positive impact on pABA production (*pabAB*, *aroE* and *aroB*), we did not consider the genetic factor with the lowest effect of the genes with negative coefficients (*aroC*) and the genetic factor with the lowest effect of the genes with positive coefficients (*pabC*) obtained from the linear model in Fig. 2C because only a mild effect of the BCD on *aroC* and *pabC* is expected in this construct. Moreover, considering that production of the strain S12 is much better than the rest of strains of the DoE, only a mild effect of changing *aroC* is expected. Four additional strains (S12-1, S12-2, S12-3, and S12-4) were constructed with bicistronic designs controlling the expression of *pabAB*, *aroB*, *aroE* or *aroB* and *aroE* using the best producer strain, S12, as background (Fig. 3B; Table 2). Controlling expression of *aroB* with a bicistronic design resulted in a statistically significant increase in production of 25.2% compared to S12 with a *p*-value of 0.023 determined by two-tailed unpaired t-test, a final titer of 232 mg/l and a yield on glucose of 0.024 mol/mol. These results suggest that translation initiation of *aroB* is a rate-limiting step for pABA production in *P. putida.* In addition, controlling expression of *aroB* with BCD2 in strain S12-4 resulted in an increase of 78% in product titer normalized to culture density compared to S12 with *p*-values < 0.001 determined by two-tailed unpaired t-test and a final product titer normalized to culture density of 85 mg/OD_600nm_ (Figure S5).

**Table 1:** Results from the two-tailed unpaired t-test analysis for the comparison between strains with mild over-expression and basal expression from genome

ID	Genes of study		Strain comparison		*P*-value	Significance
			Mild over-expression	Basal expression		
			Titer (mg/l)			
S2	*pabC-aroG* ^*D146N*^ *-aroB-aroA-aroC*		41.05	17.05	0.010	*
S4	*aroG*^*D146N*^ *-aroB-aroQ-aroE-aroC*		51.43	7.24	$\:{1.8x10}^{-5}$	***
S8	*aroB-aroE-aroK-aroA-aroC*		64.04	0	$\:{1.9x10}^{-7}$	***
S10	*pabC- aroG*^*D146N*^ *-aroE-aroQ*		15.83	6.33	0.033	*
S12	*aroG*^*D146N*^ *-aroQ-aroK-aroA*		186.21	112	0.001	**
S14	*pabC-aroQ-aroE-aroA-aroC*		111.17	12.64	$\:{7.2x10}^{-7}$	***

Titer is the average of three biological replicates. ID: identifier

**Table 2:** Results from the two-tailed unpaired t-test analysis for the comparison between the top producer strain from the Plackett Burmann design and bicistronic design strains

Strain comparison				*P*-value	Significance
Top producer strain		Bicistronic design strains			
Identifier	Titer (mg/l)	Identifier	Titer (mg/l)		
S12	185.40	S12-1	104.46	0.118	ns
		S12-2	228.10	0.007	**
		S12-3	165.98	0.523	ns
		S12-4	232.08	0.023	*

Titer is the average of three biological replicates. ns: not significant

## Discussion

Adjusting gene expression is one of the required steps to optimize chemical production in cell factories [17, 20, 38]. However, in order to consider possible synergies among genes, numerous strains that vary in the over-expressed genes must be constructed. In this study, we used a Plackett-Burman design to screen for the effect of nine genes involved in pABA production. Considering only two over-expression levels per gene, testing all possible combinations of mild and high over-expression would require the construction of 512 strains. Instead, the DoE design proposes the construction of 16 strains (3% of all the combinations). We assumed that the level of gene expression is determined by the plasmid copy number and the promoters used. For example, if the construct has a higher copy number and the promoters are strong, it is assumed that high expression of the genes will follow (and viceversa). This simplification provides a more efficient library sampling to understand the behavior of the studied pathway. However, unexpected synergies among these variables can limit the outcome of the DoE. Even though only 14 of the 16 strains could be constructed after repeated attempts, identifying the effect of each of the over-expressed genes on pABA production was possible by training a linear model on the production data, showcasing the robustness of the proposed design to missing data. Plackett and Burman [19] prescribed a contrast-based approach for data analysis. Here we have followed a compatible method [14]. In the classical method, the effect of each factor is estimated as the difference in the average response between the high (+ 1) and low (− 1) levels. This is, in fact, equivalent to the estimation of coefficients in a linear model when the design matrix is coded using + 1/−1 levels, as was the case in our analysis. The use of linear regression extends the original approach as it enables statistical testing of the significance of model coefficients, using ANOVA and Bonferroni correction, and assessment of model fit (e.g., adjusted R²).

*P. putida* is a microbial host with the ability to sustain high-levels of NADPH relevant for the production of shikimate-derived compounds [23]. This host has been engineered for the production of many relevant shikimate-derived compounds including para-hydroxybenzoic acid [25] ortho-aminobenzoic acid [39] anthranilate [40] and muconic acid [41]. However, optimizing the shikimate pathway in this microorganism remains a challenge because of their multiple interactions [2, 25, 42]. While studies focused on improving the efficiency of the shikimate pathway generally consider specific genes and one overexpression level [2] we explored all genes in the shikimate pathway under different overexpression levels. While over-expression of all the shikimate pathway genes, *pabAB* (heterodimer complex) and *pabC* is required to obtain higher pABA titers, we show that different overexpression strengths per gene are optimal. As a result of reducing gene over-expression to native expression levels in *aroA*, *aroK*, *aroQ*, and *aroG*^D146N^, we observed a statistically significant decrease of 39.9% in pABA production (*p*-value = 0.001) in the top producer strain S12 (light green bar in Fig. 3A), indicating that a low over-expression of these genes is important to achieve a higher pABA titer than basal expression from the genome. We observed that a mild over-expression of shikimate pathway related genes (light green cells in Fig. 3A) is required in order to obtain higher pABA production levels compared to the basal expression (white cells in Fig. 3A) in the strains S4, S8, and S12. While mild over-expression of *aroA*, *aroQ*, *aroG*^D146N^, and *aroC* is needed, higher over-expression of *pabABC*, *aroE*, and especially *aroB* is beneficial. In this way, the proposed approach can efficiently identify production bottlenecks caused by insufficient gene expression. Notably, although the expression of feed-back resistant variants of *aroG* is generally considered beneficial for production, high over-expression of this gene has a negative impact on pABA production in *P. putida*, probably due to an excessive expression level of this *aroG* variant that surpass the optimal range, leading to diminished pABA titers. In contrast, and as previously reported for muconic acid production in *P. putida* [41] and violacein production in *E. coli* [10] expression of *aroB* is identified as limiting.

DoE and linear modeling enabled us to identify target genes for rational engineering. Consequently, we intended to further improve pABA production by expanding the design space using Bicistronic Designs on the target genes with the highest positive impact (*pabAB*,* aroB*,* and aroE*). The functionality of the bicistronic design (BCD2) has been successfully demonstrated in *P. putida* KT2440, increasing gene expression by 60% compared to the construct without this decoupling element [36]. In agreement with the modeling outcomes, an apparent increase in expression of *aroB* using a bicistronic design resulted in a 25.2% increased pABA titer up to 232.1 mg/l. However, controlling expression with a bicistronic design also affected the growth of the strains with bicistronic designs, a phenotype not observed in the original library (Figure S1 and S3). Although growth was affected in the strain S12-4, controlling the expression of *aroB* with the BCD2 resulted in a higher pABA titer normalized to culture density (mg/OD_600nm_) compared to S12, suggesting that the incorporation of this element is beneficial to enhance product yield relative to biomass (Figure S5). Since strains S12-2 and S12-4 outperformed strain S12, future gene expression optimization efforts could explore increasing the expression levels of the designated low-expression plasmids in these strains, guided by the insights from Fig. 3A. This would enable to determine whether elevating expression of the genes under the low-expression control could further enhance pABA production in strains S12-2 and S12-4. Additionally, since our results identified *aroB* as a limiting factor on pABA production following the introduction of the bicistronic design element, a new strain could be generated using strain S12-4 as a baseline to assess whether increasing the expression of *aroB* would enhance pABA production. The reported titers are competitive compared to results obtained with other organisms such as yeast reaching 215 mg/l^26^. However, higher titers have been obtained in *E. coli* and *Corynebacterium glutamicum*, 4.8 g/l and 43 g/l, respectively, using rich media in fed-batch cultures [34, 43]. We acknowledge that the titers achieved in *E. coli* and *Corynebacterium glutamicum* significantly surpass those obtained in this study. However, our objective was not to surpass previously reported maximum titers of pABA, but rather in illustrating how DoE can be applied for systematically identifying bottlenecks within the shikimate pathway. Our work presents a rational method to identify and mitigate bottlenecks that limit the biosynthesis of shikimate-derived products. Improving the efficiency of the shikimate pathway is important, as it serves as a central route to metabolic diversity [44]. This seven-step pathway, converts primary carbon precursors into aromatic amino acids, which are then used to generate a wide array of natural products with significant industrial, pharmaceutical, and agricultural applications [44]. Our findings contribute a strategy for overcoming metabolic constraints in the shikimate pathway. Moreover, the optimization of the shikimate and pABA biosynthesis pathway can be leveraged to improve the availability of precursors from primary metabolism towards the biosynthesis of secondary metabolites that are often produced in trace amounts due to insufficient supply of substrate. Beyond the optimization of gene expression, *pabABC* gene has a significant impact on pABA production and more efficient heterologous genes could improve the titers obtained with *P. putida* [34, 43, 45, 46]. Besides, strategies such as improving precursor supply, optimizing production conditions, and deleting genes from competing pathways could further improve the strain performance [25, 45]. In addition, the use of fed-batch cultivations as part of the bioprocess optimization is an approach expected to yield higher product titers compared to the short batch cultivations in falcon tubes used in this study [47].

In the light of emerging tools for pathway optimization, DoE is complementary to strategies based on machine learning, especially when the capacity to build and screen strains is limited. The data generated with DoE designs can be used to train more complex machine learning models [18]. Despite the potential of these complex models, they are limited by the difficult interpretation of their predictions. Here, we show the value of linear regression models to directly relate model outcomes to engineering strategies (e.g. positive model coefficients suggest the need of higher gene expression).

In conclusion, this study demonstrates an effective strategy to explore the metabolic pathway design for overproduction of pABA by manipulating the expression levels of *pabABC* and all shikimate pathway genes. We could identify *aroB* expression as a relevant limiting step in the accumulation of this aromatic compound in *P. putida*. Considering that combinatorial metabolic engineering is a requirement for strain optimization, we demonstrate the utility of this approach for the optimization of metabolite production and the identification of bottlenecks that inform rational cell factory design.

## Supplementary Information

Below is the link to the electronic supplementary material.

Supplementary Material 1

Supplementary Material 2

Supplementary Material 3

Supplementary Material 4

## Data Availability

Data and materials are provided within the manuscript or supplementary information files.
