# Supplementary material for: Combinatorial engineering pinpoints shikimate pathway bottlenecks in para-aminobenzoic acid production in Pseudomonas putida
Source: J Biol Eng. 2025 Sep 30;19:81. doi: 10.1186/s13036-025-00553-5 (PMC12487284; doi:10.1186/s13036-025-00553-5)
Supplement: Supplementary file 4 — Supplementary Material 4 [file 13036_2025_553_MOESM4_ESM.docx]

**Supplementary materials**

Combinatorial engineering pinpoints shikimate pathway bottlenecks in para-aminobenzoic acid production in *Pseudomonas putida*

Marco A Campos-Magaña, Sara Moreno-Paz, Maria Martin-Pascual, Vitor AP Martins dos Santos, Luis Garcia-Morales, Maria Suarez-Diez.

**Supplementary method 1. RT-qPCR**

An overnight preculture of *P. putida* S1 and S16 were used for reinoculation 25 ml of fresh minimal M9 medium supplemented with 70 mM glucose, kanamycin (50 μg/mL) and gentamycin (10 μg/mL), respectively, reaching a OD_600nm_ of 0.3_._ The cultures of *P. putida* were grown at 30°C until an OD_600nm_ of 0.5-1 was reached. 15 mL of culture were harvested at room temperature and 500g for 5 min. RNA isolation was performed using Maxwell® RSC simplyRNA Cells Kit. The purified RNA was measured in a Nanodrop spectrophotometer and used for cDNA synthesis following the RevertAid First Strand cDNA Synthesis Kit. RT-qPCR was performed using iQ SYBR Green Supermix from BioRad. The house-keeping gene used to measure the relative expression was the RNA polymerase sigma factor *rpoD* of *P. putida*. The primers used to amplify the house-keeping gene as well as all target genes are listed in Table S1. The annealing temperature was set to 60°C and extension of 30 s. The RT-qPCR was performed in a Corbett Thermal Cycler RG3000 (Life Science). Pfaff1 method was used to calculate relative gene expression levels between a target gene in comparison with a reference gene (house-keeping gene) and amplification efficiencies were calculated by performing 10-fold dilutions.

**Supplementary method 2. Function from the FrF2 R package used to generate 16-run design, 9 factors, and 0 dummies**


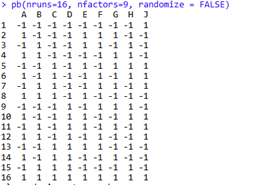


Design information with dummy variables


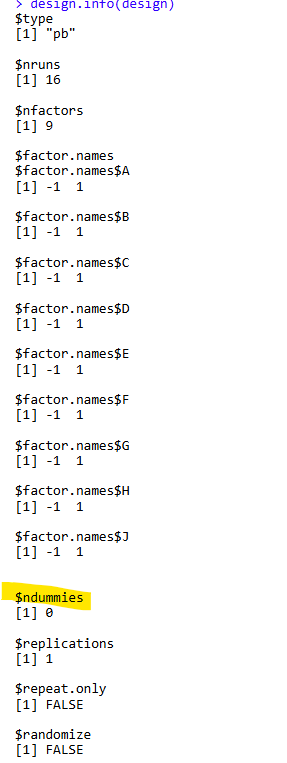


|  | **Coefficient** | **p-value** | **Corrected p-value** |
| --- | --- | --- | --- |
| **AroA** | **-0,21218** | **1,30E-14** | ******* |
| **AroQ** | **-0,2018** | **4,10E-15** | ******* |
| **AroK** | **-0,10999** | **1,20E-08** | ******* |
| **AroG^D146N^** | **-0,08947** | **3,20E-06** | ******* |
| **AroC** | **-0,07155** | **8,40E-05** | ******* |
| **pabC** | **0,08216** | **1,20E-05** | ******* |
| **AroE** | **0,11326** | **6,60E-09** | ******* |
| **AroB** | **0,13237** | **1,60E-09** | ******* |
| **pabAB** | **0,25387** | **2,00E-16** | ******* |
|  |  |  |  |
| **Adj R2** | **0,94** |  |  |

Table S1. Original Regression coefficients of the linear model.

|  | **Coefficient** | **Standard error** | **t value** | **p-value** | **Corrected p-value** |
| --- | --- | --- | --- | --- | --- |
| **AroA** | **-0,478933** | **0,027627** | **-17,336** | **2,00E-16** | ******* |
| **AroQ** | **-0,381138** | **0,023926** | **-15,930** | **7,15E-16** | ******* |
| **AroK** | **-0,197513** | **0,023926** | **-8,255** | **4,22E-09** | ******* |
| **AroG^D146N^** | **-0,233525** | **0,027627** | **-8,453** | **2,58E-09** | ******* |
| **AroC** | **-0,197683** | **0,027627** | **-7,155** | **7,10E-08** | ******* |
| **pabC** | **0,218900** | **0,027627** | **7,923** | **9,73E-09** | ******* |
| **AroE** | **0,204071** | **0,023926** | **8,529** | **2,14E-09** | ******* |
| **AroB** | **0,319325** | **0,027627** | **11,558** | **2,24E-12** | ******* |
| **pabAB** | **0,485279** | **0,023926** | **20,283** | **2,00E-16** | ******* |
| **Dummy 1** | **-0,109158** | **0,027627** | **-3,951** | **4,57E-04** | ******* |
| **Dummy 2** | **0,080296** | **0,023926** | **3,356** | **2,22E-03** | ****** |
| **Dummy 3** | **-0,009538** | **0,023926** | **-0,399** | **6,93E-01** |  |
|  |  |  |  |  |  |
| **Adj R2** | **0,9647** |  |  |  |  |

Table S2. Regression coefficients with real variables and 3 dummies.

Dummy variable coefficients

To further assess the likelihood that the observed main effects were due to random variation, we computed z-scores for each real factor coefficient using the empirical distribution of dummy variables (mean = –0.013, standard deviation = 0.097). Coefficients with |z| > 2 were considered statistically significant. Seven out of nine variables exceeded this threshold, with z-scores ranging from –4.81 to +5.13, indicating strong and non-random effects. Variables aroK and aroC had borderline significance (z ≈ –1.90), suggesting weaker but potentially meaningful contributions. This analysis reinforces that the most important observed effects are unlikely to be artifacts of noise.

**Supplementary figures**


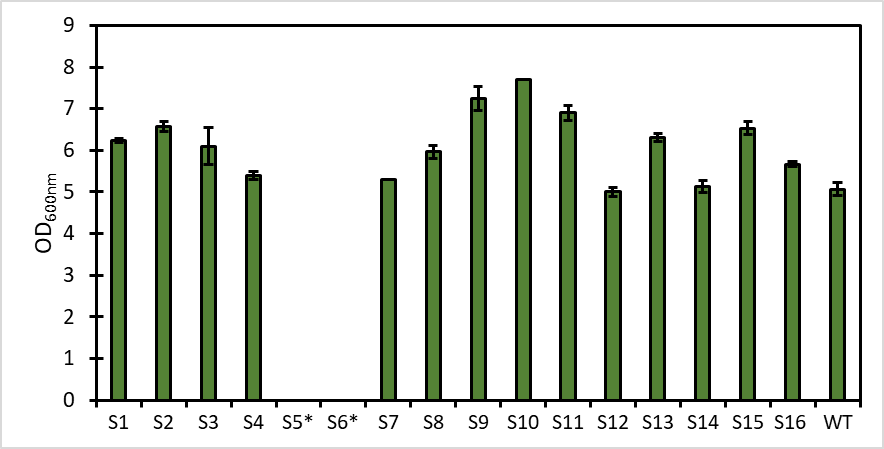


Figure S1. Growth performance measured at OD_600nm_ for strains S1 to S16 created in this study and represented in Figure 2B in the main manuscript. Strains S5 and S6 could not be constructed and therefore they are marked with an asterisk. Values are means of the biological replicates, and the error bars indicate the standard deviations of all (n=3) biological replicates.


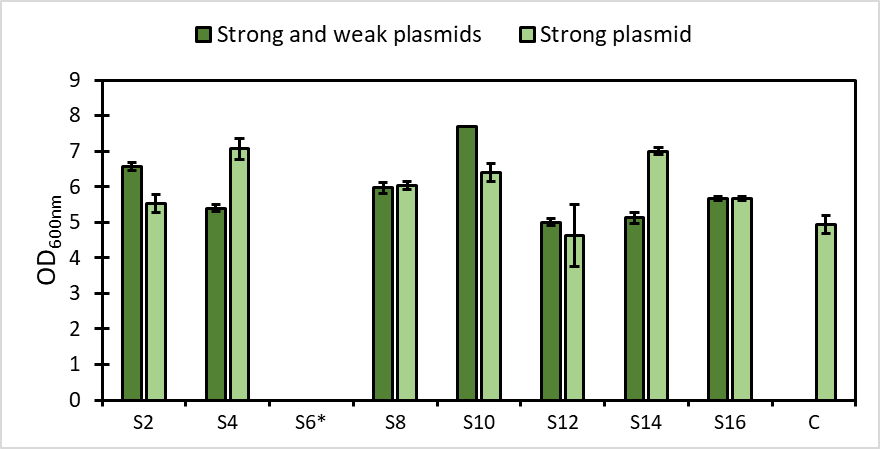


Figure S2. Growth performance measured at OD_600nm_ for strains S2, S4, S8, S10, S12, S14 under the effect of reducing gene over-expression to native expression levels for the genes in white cells in Figure 3A. Dark green bars depict strains S2, S4, S8, S10, S12, S14, and S16 from Plackett Burman design, while light green bars depicts strains S2, S4, S8, S10, S12, and S14 under the effect of reducing gene over-expression to native expression levels for the genes in white cells in Figure 3A. As a comparison, we also included a control strain C representing the effect of only high overexpression in *pabABC* genes. Values are means of the biological replicates, and the error bars indicate the standard deviations of all (n=3) biological replicates.


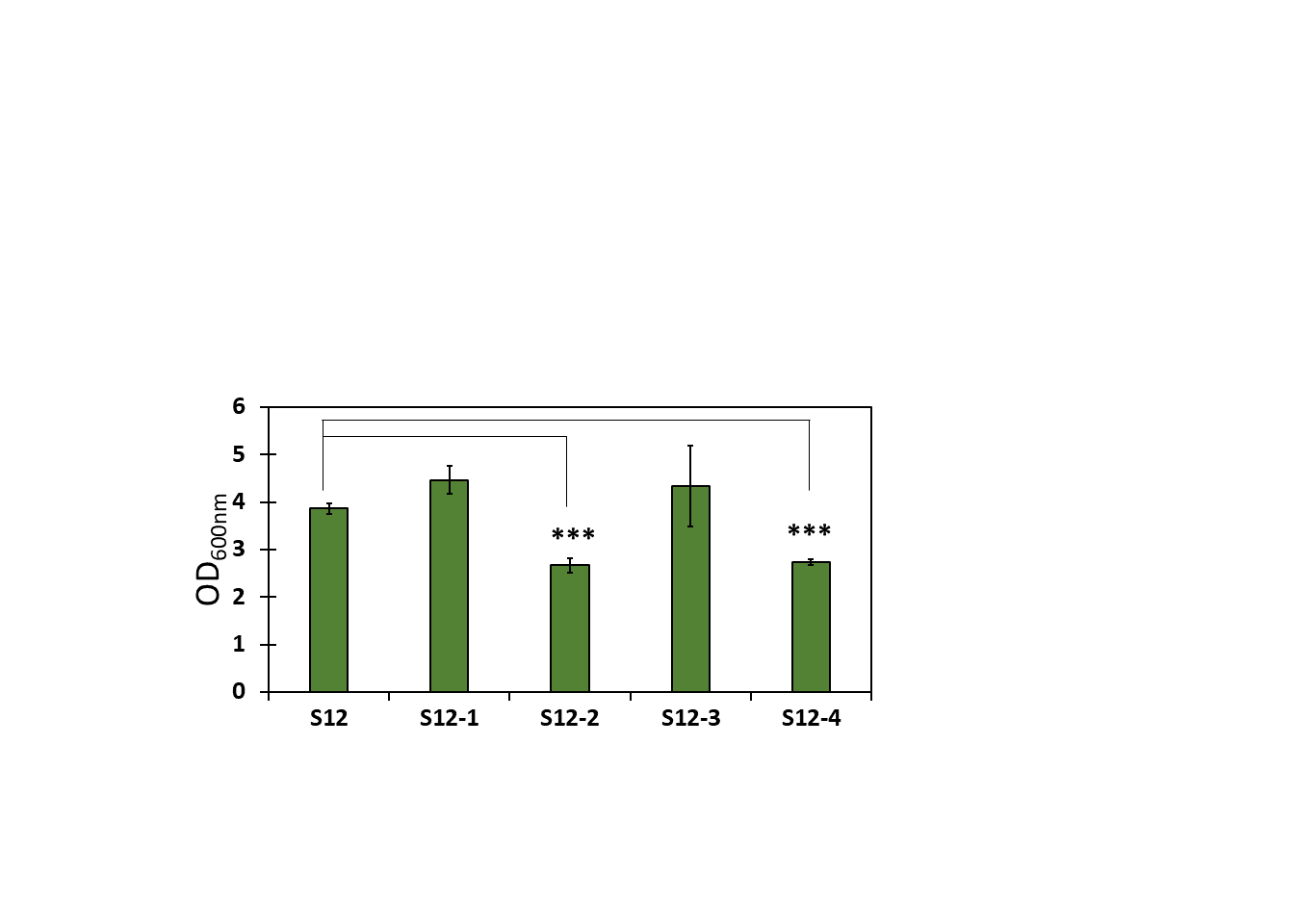


Figure S3. Growth performance measured at OD_600nm_ for strains under the effect of a bicistronic design element in genes marked in yellow in Figure 3B. T-test analysis was performed to show a significant difference between strain S12 and S12-4. Values are means of the biological replicates, and the error bars indicate the standard deviations of all (n=3) biological replicates *P*-values were determined by two-tailed unpaired t-tests. *p < 0.05; **p < 0.01; ***p < 0.001.


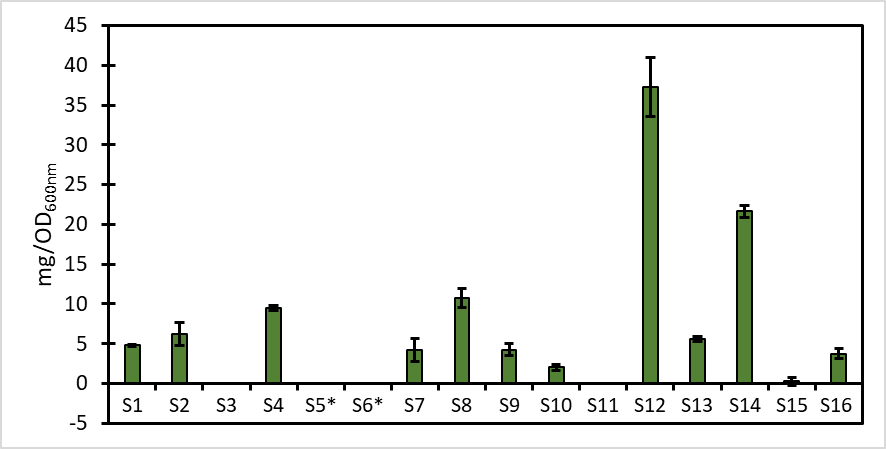


Figure S4. mg of pABA/OD_600nm_ for strains S1 to S16 created in this study and represented in Figure 2B in the main manuscript. Strains S5 and S6 could not be constructed and therefore they are marked with an asterisk. Values are means of the biological replicates, and the error bars indicate the standard deviations of all (n=3) biological replicates.


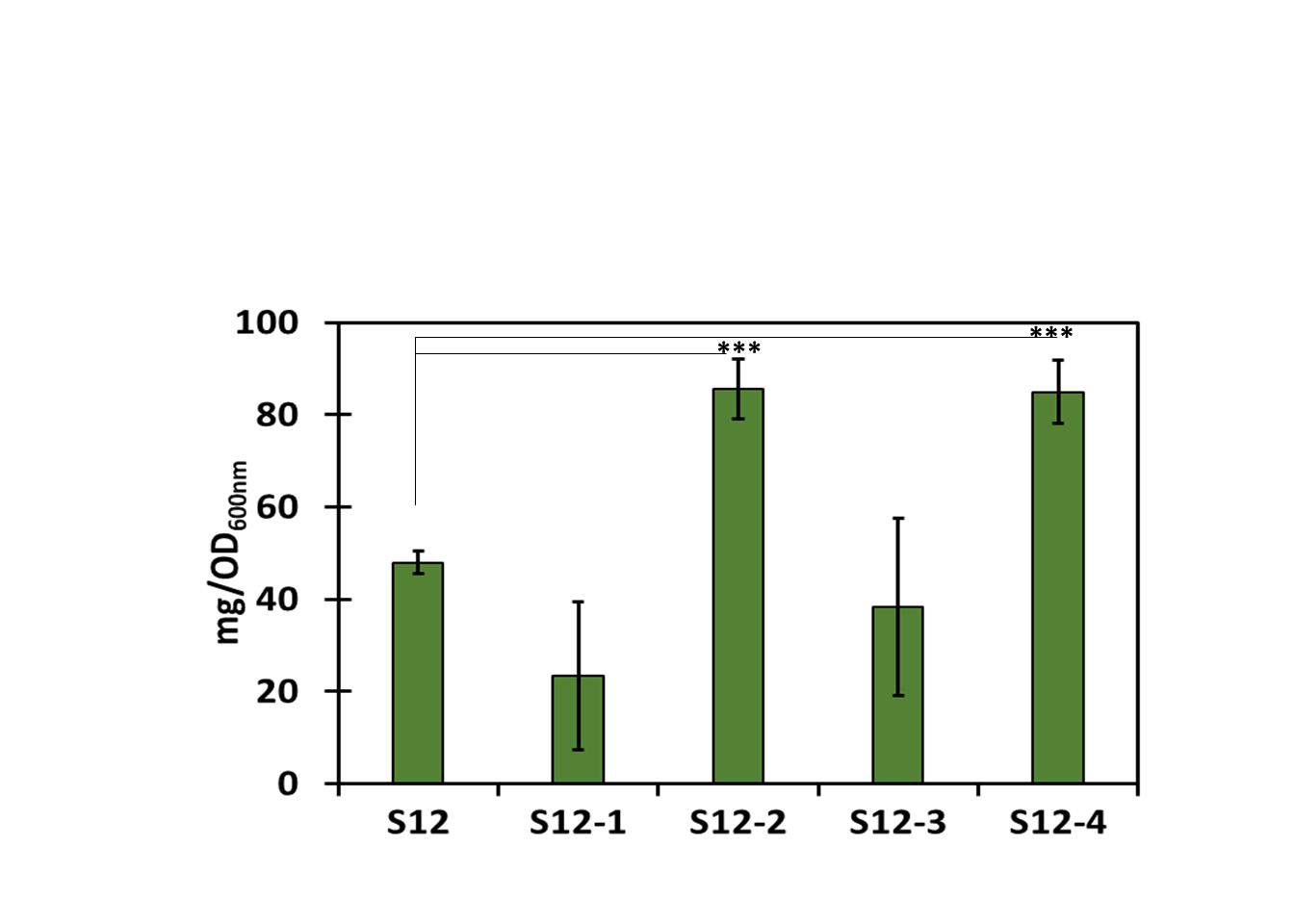


Figure S5. mg of pABA/OD_600nm_ for strains under the effect of a bicistronic design element in genes marked in yellow in Figure 3B. Values are means of the biological replicates, and the error bars indicate the standard deviations of all (n=3) biological replicates. *P*-values were determined by two-tailed unpaired t-tests. *p < 0.05; **p < 0.01; ***p < 0.001.


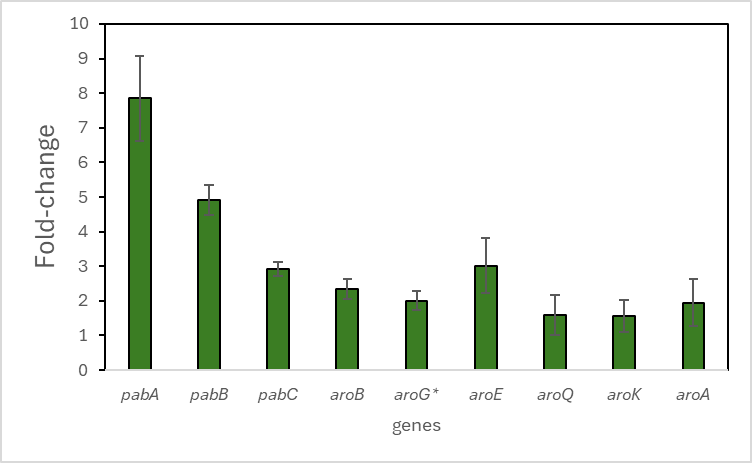


Figure S6. RT-qPCR. Gene expression differences in the strain S16 compared to strain S1 for the genes *pabA*, *pabB*, *pabC*, *aroB*, *aroG**, *aroE*, *aroQ*, *aroK*, *aroA.** indicates feed-back resistant variant aroG^D146N^. Values are means of the biological replicates and the error bars indicate the standard deviations of all (n=3) biological replicates.


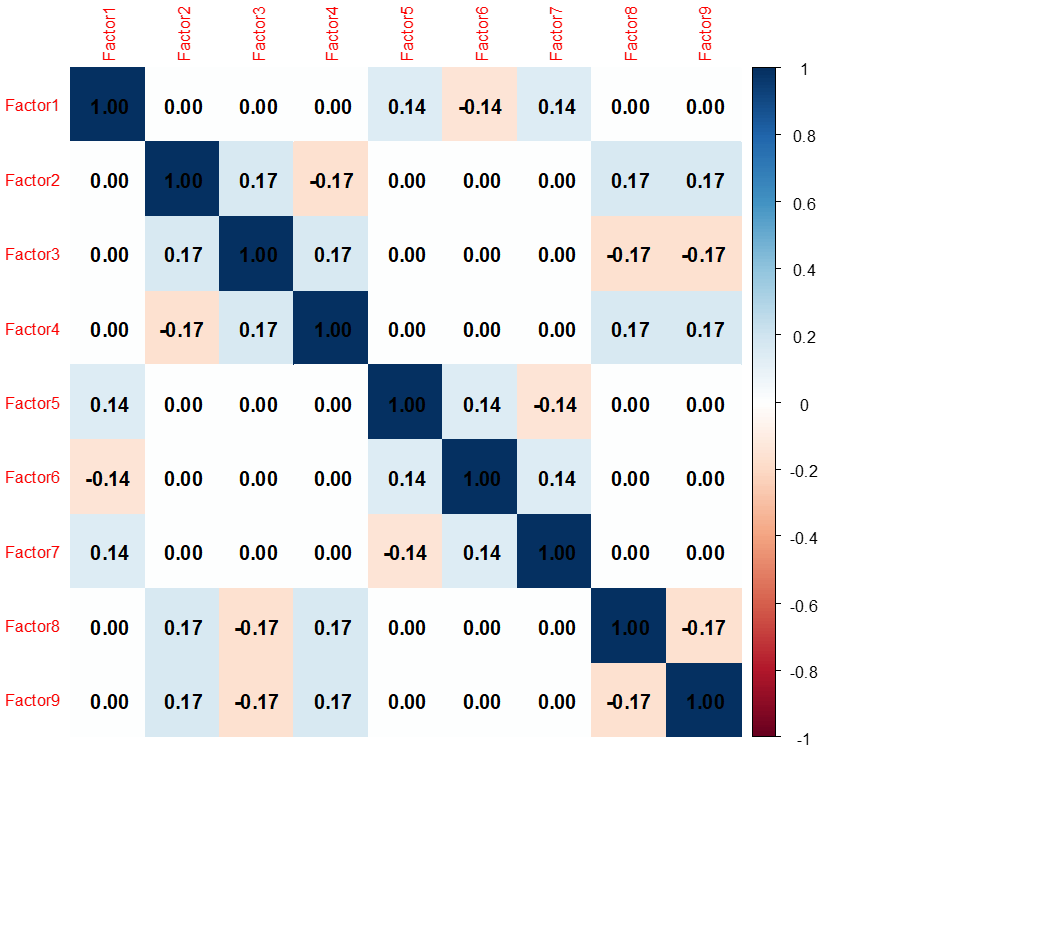


Figure S7. Correlation analysis for the 14-run matrix

The correlation analysis of the 14-run Plackett–Burman design (excluding runs 5 and 6) indicates that not all correlations are zero, thereby compromising the balanced orthogonality (Figure S7). However, the resulting 14-run design retained low pairwise correlations among factors (|r| ≤ 0.17). While this compromised the balanced orthogonality, the main effects can still be estimated with minimal bias for the purpose of screening.
